# From hormonal immunomodulation to glioblastoma therapy: the emerging role of Ouabain

**DOI:** 10.3389/fimmu.2025.1657671

**Published:** 2025-11-07

**Authors:** Arthur Gomes de Andrade, Deyse Cristina Madruga Carvalho, Daniel Wilson Arruda Magalhaes, Aline Cavalcanti de Queiroz, Magna Suzana Alexandre-Moreira, Sandra Rodrigues-Mascarenas, Luiz Henrique Agra Cavalcante-Silva

**Affiliations:** 1 Biotechnology Center, Federal University of Paraíba, João Pessoa, Paraíba, Brazil; 2 Laboratory of Pharmacology and Immunity, Institute of Biological Sciences and Health, Federal University of Alagoas, Maceió, Brazil; 3 Medical Sciences and Nursing Complex, Federal University of Alagoas, Arapiraca, Alagoas, Brazil

**Keywords:** cancer, cardiotonic steroid, tumor microenvironment, GBM, cytotoxicity

## Abstract

Glioblastoma (GBM) is the most aggressive primary brain tumor in adults, characterized by rapid proliferation, diffuse infiltration, and resistance to conventional therapies. Despite advances in surgery, radiotherapy, and chemotherapy, the prognosis remains dismal, with median survival rarely exceeding 15 months. The immunosuppressive and heterogeneous tumor microenvironment (TME), along with profound tumor-intrinsic resistance mechanisms, contributes significantly to treatment failure. Cardiotonic steroids (CTS), such as ouabain, have recently gained attention for their pleiotropic effects beyond Na^+^/K^+^-ATPase inhibition, including modulation of intracellular signaling, induction of cell death, and immune regulation. In GBM, ouabain has been shown to reduce tumor cell viability, impair migration, disrupt angiogenesis, and alter different signaling pathways. Although direct evidence of ouabain’s effects on the GBM immune microenvironment is limited, findings from other models suggest that it can modulate both innate and adaptive immune responses, affecting T cells, regulatory T cells, dendritic cells, monocytes, and NK cells. While previous reviews have explored the anticancer and pharmacological aspects of cardiotonic steroids, the immunological dimension of ouabain’s activity remains underrepresented. This review integrates current evidence on ouabain’s dual actions in tumor biology and immune regulation, emphasizing its emerging therapeutic potential and the need for deeper investigation within high-grade glioma models.

## 1 Introduction

Glioblastoma (GBM) is the most aggressive primary brain tumor, characterized by rapid proliferation, diffuse invasion into surrounding brain tissue, and resistance to conventional therapies (1, 2). Standard treatment typically involves maximal surgical resection followed by radiotherapy and chemotherapy [i.e., temozolomide (TMZ)] (3). However, despite their widespread use, the success rate of treatment remains low due to intrinsic and acquired resistance mechanisms. Tumor cells often develop resistance through O6-methylguanine-DNA methyltransferase (MGMT) expression, DNA repair pathways, and metabolic adaptations that allow survival even with cytotoxic drugs (4–6). As a result, median survival for GBM patients remains approximately 12–15 months (7, 8), underscoring the urgent need for novel therapeutic approaches.

The highly heterogeneous and immunosuppressive tumor microenvironment (TME) in GBM contributes to tumor progression and therapeutic resistance by impairing anti-tumor immune responses and promoting tumor-supportive interactions (9, 10). Non-immunological components such as the vasculature, cancer stem cells (CSCs), astrocytes and neurons actively contribute to sustaining the tumor microenvironment, providing structural support, modulating metabolic exchanges, and influencing tumor plasticity (11–14). Among the main immune components in the GBM TME are tumor-associated macrophages (TAMs), regulatory T cells (Tregs), and myeloid-derived suppressor cells (MDSCs), all of which actively suppress tumor-infiltrating lymphocytes (TILs) and natural killer (NK) cells (15, 16), thereby facilitating immune evasion and challenging the use of immunotherapies (17, 18).

One potential approach for therapeutic intervention involves targeting ion homeostasis in both cancer and immune cells (19–21). Ouabain, a cardiotonic steroid known for its ability to inhibit the ubiquitous ion pump Na^+^/K^+^-ATPase at higher concentrations, has emerged as a potential modulator of cellular signaling beyond its classical role in cardiac function (22, 23). At lower concentrations, ouabain induces calcium oscillations leading to the activation of important signaling pathways involved in cellular homeostasis and function (23–26). Different studies in tumor models suggest that ouabain can influence tumor cell proliferation, apoptosis, and migration, including in GBM cell lines (26–32). Additionally, ouabain can potentially modulate immune cell activity in the TME, shifting the balance from an immunosuppressive to an anti-tumor immune response (33, 34).

Given ouabain’s dual role in cancer and immune cell modulation, understanding its effects in the GBM TME could offer new insights into its therapeutic potential. Although few comprehensive reviews have examined CTS as anticancer agents, most have centered on their cytotoxicity, structure–activity relationships, or general pharmacology across multiple malignancies (35–37). In contrast, the present review provides a focused analysis of ouabain, emphasizing not only its established effects on GBM cell survival and signaling but also its underexplored immunomodulatory properties. Given the absence of studies directly assessing ouabain in the GBM TME, we draw on findings from other tumor and immune models to infer how ouabain might reshape immune–tumor dynamics in GBM. This integrative perspective bridges tumor-intrinsic and immune-related mechanisms, offering a framework to understand ouabain’s multifaceted therapeutic potential in high-grade gliomas.

## 2 GBM architecture: from the core to the neighborhood

Glioblastoma is characterized by profound cellular heterogeneity and a complex genomic landscape, with frequent alterations in pathways regulating proliferation, apoptosis, metabolism, and DNA repair, all of which contribute to therapeutic resistance and complicate effective disease management (38). Beyond its intrinsic resistance to treatment, GBM’s malignant behavior is also shaped by a complex TME that includes both tumor-intrinsic and tumor-extrinsic components (39). A comprehensive understanding of this integrated tumor microenvironmental network is essential for identifying mechanisms of GBM progression and uncovering novel therapeutic targets.

At the tumor core, GBM cells exhibit substantial genetic and phenotypic heterogeneity, often driven by mutations in critical regulators such as IDH1/2, EGFR, TP53, and PTEN (40). The presence of glioma stem-like cells (GSCs), typically marked by CD133 expression, contributes significantly to tumor persistence and therapeutic failure, as these cells exhibit enhanced capacities for self-renewal, resistance to apoptosis, and evasion of immune surveillance (41). GSCs also actively remodel the microenvironment by secreting factors such as CSF-1 and CCL2, which recruit monocytes and promote their polarization into tumor-supportive macrophages (42, 43).

The TME of GBM exhibits distinct features compared to other solid tumors, owing primarily to its localization within the CNS and its unique immune and stromal composition. It is broadly categorized into immunological and non-immunological compartments, both of which interact extensively with tumor cells to support disease progression (11). Non-immunological components include a highly disorganized and permeable vasculature, a rigid and biochemically active extracellular matrix (ECM), reactive astrocytes, and neurons that undergo functional reprogramming in response to tumor-derived signals (12, 44–47). The abnormal vasculature contributes to elevated interstitial fluid pressure and regional hypoxia, which in turn stabilizes HIF-1α, an important transcriptional regulator that promotes angiogenesis, metabolic reprogramming, and cellular adaptation to oxygen deprivation, including a shift toward aerobic glycolysis consistent with the Warburg effect (48, 49).

Neurons and astrocytes, once considered passive bystanders, are now recognized as active contributors to tumor progression (13). Neuronal activity has been shown to directly influence tumor growth through the activity-dependent release of neuroligin-3 (NLGN3), which promotes GBM cell proliferation via activation of the PI3K-mTOR signaling pathway (50, 51). Astrocytes similarly contribute to tumor maintenance by secreting a variety of mitogenic and trophic factors, including cytokines and growth factors that enhance glioma cell survival, invasion, and therapy resistance (14, 52).

The immunological compartment of the GBM TME is dominated by immunosuppressive mechanisms that hinder effective antitumor responses (9). TAMs, which may arise from resident microglia or peripheral monocytes, are the most abundant immune cells in GBM (53). These cells are often polarized to an M2-like phenotype, characterized by high expression of IL-10, TGF-β, and MMPs, contributing to tissue remodeling, angiogenesis, and immunosuppression. Notably, bone marrow–derived TAMs constitute approximately 85% of the macrophage population in GBM and exhibit particularly tumor-promoting features (54).

T cell infiltration in GBM is often sparse and functionally compromised (55). The T cell compartment is enriched in Tregs, which suppress effector responses, while cytotoxic CD8^+^ T cells frequently exhibit an exhausted phenotype, marked by the upregulation of multiple immune checkpoint receptors, including PD-1, TIM-3, LAG-3, and CTLA-4 (56–58). This dysfunctional state is associated with reduced proliferative capacity, diminished cytokine production, and impaired cytotoxic function (59). The extent and composition of T cell infiltration appear to be influenced by IDH mutation status. IDH-wild-type tumors tend to display greater immune cell infiltration and elevated immune checkpoint expression, whereas IDH-mutant GBMs are typically characterized by a more immunologically quiescent microenvironment with limited lymphocyte presence and reduced immunogenicity (60).

Beyond the limited and dysfunctional T cell compartment, innate immune cells constitute a substantial portion of the GBM TME and play diverse, often immunosuppressive roles. NK cells can recognize and eliminate GSCs, and their presence has been associated with favorable outcomes in specific glioma subtypes (61–63). However, in this scenario, NK cell function is frequently impaired due to chronic activation, exposure to immunosuppressive cytokines, and the expression of inhibitory ligands such as B7-H6 on tumor cells, which contribute to NK cell exhaustion (64, 65). Moreover, NK cells interact with DCs through chemokines such as XCL1 and FLT3L, promoting the recruitment of conventional type 1 dendritic cells (cDC1) that are critical for cross-presentation of tumor antigens (66, 67). Despite this crosstalk, DCs in high-grade gliomas often exhibit functional deficiencies, particularly in the context of IDH-mutant tumors, where impaired antigen presentation further compromises antitumor immunity (68).

Additional myeloid populations, including neutrophils and MDSCs, further contribute to immune evasion and tumor progression. Neutrophils can exert protumor effects through the secretion of pro-inflammatory and pro-angiogenic mediators such as S100A4 and IL-8, and by forming neutrophil extracellular traps (NETs) that activate NF-κB signaling in glioma cells (69–71). Their role in GBM is highly context-dependent, as they exhibit considerable phenotypic plasticity, shifting between tumor-promoting (N2-like) and potentially tumor-inhibiting (N1-like) states depending on microenvironmental cues (72). Neutrophils actively migrate into the GBM TME primarily from the skull and vertebral bone marrow, utilizing specialized cranial bone channels and intracranial lymphatic vessels. Once within the TME, tumor-associated neutrophils (TANs) preferentially localize in necrotic tumor cores and undergo functional reprogramming, sustaining tumor progression through the release of NETs and immunosuppressive mediators (72, 73). MDSCs, which are commonly expanded in GBM, suppress T cell proliferation and cytokine production through arginase-1 activity, nitric oxide production, and the release of immunosuppressive cytokines, thereby reinforcing the profoundly suppressive immune landscape characteristic of this malignancy (74–76).

Altogether, the TME of GBM is a tightly regulated network in which tumor cells, immune components, and neural elements interact to create a profoundly immunosuppressive and tumor-supportive niche. This complexity not only contributes to therapeutic resistance but also poses a significant challenge for the development of effective immunotherapies. Targeting this microenvironment, whether by reprogramming TAMs, enhancing NK and T cell function, or disrupting tumor-neuron crosstalk, represents a promising frontier in GBM research.

## 3 GBM modulation by ouabain

Over the past decade, there has been increasing attention on CTS for their emerging antitumor properties (35). Originally recognized for their role in cardiovascular therapy by inhibiting the Na^+^/K^+^-ATPase pump, CTS have demonstrated pleiotropic effects in cancer models, including the modulation of cell proliferation, apoptosis, angiogenesis, and immune responses (36). Among these compounds, ouabain stands out because of its well-characterized molecular targets and its capacity to influence intracellular signaling pathways in a concentration-dependent manner (23, 77). Although the antitumor potential of CTS has been investigated in several malignancies (37, 78), evidence specifically linking ouabain to GBM is beginning to emerge.

Different studies have explored the multifaceted effects of ouabain in GBM cells. By interacting with the Na^+^/K^+^-ATPase, ouabain influences intracellular ion balance; however, its biological impact extends well beyond this canonical function, affecting key signaling pathways and cellular processes that are central to GBM pathophysiology.

One of the most consistent findings across *in vitro* studies is the capacity of ouabain to impair GBM cell viability. In both TMZ-sensitive and TMZ-resistant glioma cell lines, ouabain reduces proliferation and promotes cell death through mechanisms that involve apoptosis, necrosis, or a hybrid mechanism (30, 79), as well as suppressing tumor growth *in vivo* (80). This cytotoxic effect appears to be mediated, at least in part, by mitochondrial pathways, with activation of pro-apoptotic proteins such as Bak and an increase in reactive oxygen species (ROS) (30). Notably, the study by Yan et al. (2015) revealed that ouabain-induced ROS production is regulated via the ERK-p66Shc pathway, suggesting a specific molecular cascade through which oxidative stress is triggered (32). This ROS generation contributes to mitochondrial dysfunction and ultimately to apoptotic cell death (81), providing a mechanistic explanation for the antitumor activity observed.

In addition to its pro-apoptotic properties, ouabain has been shown to impair GBM cell migration and invasion, likely through the disruption of the Akt/mTOR signaling axis, as observed in U-87MG cells (31). This pathway is crucial not only for cell movement but also for metabolic adaptation and resistance to stress (82, 83), implying that ouabain may impair GBM cells’ ability to survive in its hostile microenvironment (84). Interestingly, while most studies report a downregulation of Akt signaling following ouabain exposure, Weidemann et al. (2023) demonstrated a concentration-dependent modulation in TMZ-resistant T98G cells, with a marked upregulation of phosphorylated Akt at 0.1 µM and a significant downregulation of pan-Akt at 1 µM (30). These divergent responses suggest a context- and dose-dependent effect of ouabain, potentially reflecting adaptive signaling mechanisms in resistant GBM phenotypes.

Supporting this, Hsu et al. (2015) demonstrated that ouabain induces cytosolic acidification and downregulates phosphorylated Akt in GBM cells, further promoting mitochondrial apoptosis through Bak activation. Although less potent than Epi-reevesioside F, a cardiac glycoside evaluated in the study, ouabain exhibited similar mechanisms of action, reinforcing the role of Na^+^/K^+^-ATPase inhibition in disrupting metabolic homeostasis and triggering cell death in GBM (85).

The interaction of ouabain with angiogenesis also adds another dimension to its therapeutic potential. GBM relies heavily on the formation of abnormal vasculature to sustain its rapid growth, largely driven by hypoxia-inducible factors such as HIF-1α. Ouabain has been shown to inhibit VEGF-A–induced angiogenesis *in vitro*, with submicromolar potency in HUVEC spheroids, and to suppress HIF-1α expression, which could limit the tumor’s capacity to establish and maintain blood supply (30). This anti-angiogenic effect aligns with ouabain’s ability to interfere with pro-survival and pro-growth pathways and reinforces its potential role in targeting the GBM microenvironment.

On a broader scale, ouabain treatment in GBM culture has also been associated with changes in Na^+^/K^+^-ATPase subunit expression, as shown in early studies demonstrating marked upregulation of α1 and α3 isoforms following exposure (86). These alterations may represent compensatory responses but also underscore the role of Na^+^/K^+^-ATPase as more than a passive ion transporter, serving instead as a signaling platform that interacts with oncogenic networks.

This understanding of ouabain’s molecular targets has prompted interest in identifying tumor-specific markers of sensitivity. Recent pan-cancer data also explore the potential selectivity of ouabain in glioma. Zhang et al. (2024) identified a negative correlation between PLAT expression, a venous thromboembolism-associated gene upregulated in gliomas, and ouabain sensitivity, suggesting that tumors with high PLAT levels may be more susceptible to ouabain’s cytotoxic effects (87). Although not experimentally validated in GBM models, these findings offer a rationale for further exploring ouabain responsiveness in molecularly stratified glioma subtypes.

In addition to its direct cytotoxic and signaling-modulatory effects on GBM cells, ouabain has also been implicated in regulating immune cell activity across multiple biological systems. Although tumor-intrinsic effects of ouabain are increasingly documented, its impact on the tumor immune microenvironment (TIME) in GBM remains poorly defined. Despite direct evidence of ouabain’s immunomodulatory effects within the GBM microenvironment is limited, drawing on findings from other tumor models and immune studies provides a preliminary framework to hypothesize how ouabain might influence glioma-associated immune cells.

CTS can modulate antitumor immunity beyond their canonical cytotoxic roles. These compounds can induce immunogenic cell death (ICD) characterized by calreticulin exposure, ATP and HMGB1 release, and secretion of HSP70/90, thereby promoting dendritic cell activation and antigen presentation. Mechanistically, ICD triggered by CTS has been linked to activation of the PERK/eIF2α/ATF4/CHOP pathway, connecting endoplasmic reticulum stress to adaptive immune priming (88).

In addition, these molecules can reshape the TIME by modulating checkpoint and cytokine signaling pathways. Ouabain and related compounds can influence the expression of PD-L1 and other immunoregulatory molecules, as well as enhance antigen-presentation machinery, suggesting a context-dependent dual role in immune evasion and sensitization to checkpoint blockade (34). It has also been demonstrated that digoxin alters myeloid cell composition and promotes early inflammatory remodeling, while Na^+^/K^+^-ATPase inhibition by CTS can activate the NLRP3 inflammasome and IL-1β release, fostering local immune activation (89, 90). NLRP3 inflammasome activation is well known to play a dual role in cancer immunity, promoting both inflammatory anti-tumor responses and, in some cases, tumor progression depending on the microenvironment (91, 92). These findings indicate that cardiac glycosides may not only act as direct cytotoxins but also as immunomodulatory adjuvants capable of converting immunologically “cold” tumors into more inflamed, immune-responsive phenotypes.

Although specific data on ouabain in TIME is limited, its effects on different contexts provide insights into its broader immunomodulatory capacity. One of the first described immunomodulatory effects of ouabain was its ability to inhibit lymphocyte proliferation induced by the mitogens phytohemagglutinin and concanavalin A (93). Subsequent studies demonstrated that ouabain reduces the proliferation of CD4^+^ and CD8^+^ T lymphocytes at concentrations that do not diminish NKA activity, indicating that this immunomodulatory mechanism occurs independently of pump inhibition (94). Complementing these effects, it was demonstrated that pre-treatment of mice with ouabain, both injected and non-injected with melanoma, reduced the number of Tregs in their spleens, an effect associated with increased survival in these animals (95). Given the well-established role of Tregs in suppressing cytotoxic responses in the GBM microenvironment (58), this effect raises the possibility that ouabain could alleviate immune suppression in gliomas by modulating Treg homeostasis, as observed with other approaches (96, 97).

Ouabain also influences B lymphocyte behavior, decreasing the quantity of mature B lymphocytes in peripheral blood while increasing their presence in lymph nodes (95, 98). Although B cells are less prominent in the GBM immune landscape, tertiary lymphoid structures and activated B cell subsets have been implicated in both contexts of promoting tumor progression (99, 100) and shaping antitumor immunity (101–103), warranting further exploration of ouabain’s influence on humoral responses in glioma.

Regarding innate immunity, ouabain appears to exert dose-dependent effects on NK cells. While early *in vitro* studies suggested that NK cell cytotoxicity is largely resistant to ouabain at low concentrations (104), more recent *in vivo* data indicate enhanced NK cell activity following ouabain treatment, evidenced by increased cytotoxic potential in NK cells isolated from mice administered 0.75 mg/kg of ouabain (105). This is particularly relevant given the capacity of NK cells in targeting glioma stem-like cells (106, 107) and their association with improved outcomes in certain GBM subtypes (61, 62). In addition, neutrophil infiltration promotes glioma cell proliferation, alters cellular organization, enhances NF-κB–dependent signaling, and correlates with poor prognosis (72, 78). Thus, given the prominent role of neutrophil migration in promoting glioblastoma progression, the ability of ouabain to inhibit neutrophil infiltration (108–110) may represent a beneficial immunomodulatory effect within the GBM tumor microenvironment.

**Figure 1:**
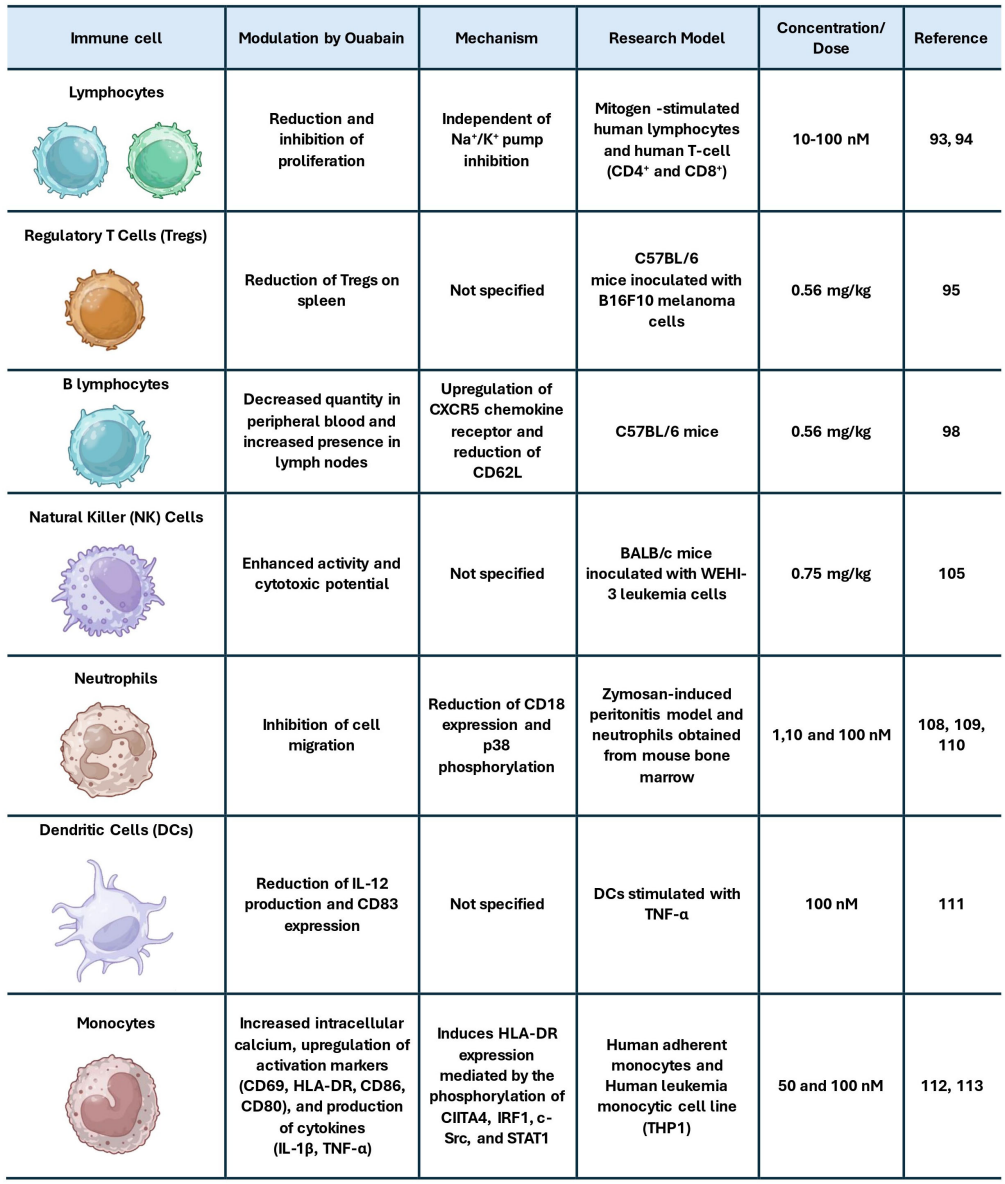
Summary of the main effects of ouabain on immune cells.

Beyond its effects previously mentioned, ouabain has also been shown to reduce IL-2 production and CD83 expression in DCs stimulated with TNF-α (111). It also promotes an increase in intracellular calcium in monocytes, along with higher expression of activation surface markers like CD69, HLA-DR, CD86, and CD80, and an increased production of cytokines such as IL-1β and TNF-α (112). Corroborating this, a recent study demonstrated that ouabain induces HLA-DR expression in monocytes, mediated by the phosphorylation of CIITA4, IRF1, c-Src, and STAT1 (113). Considering that monocyte-derived cells are the predominant myeloid population in the GBM microenvironment and that defective antigen presentation is a major barrier to effective antitumor immunity (114–116), these findings suggest that ouabain may have the potential to modulate myeloid cells toward a more immunostimulatory phenotype in gliomas. **Figure 1** summarizes and integrates the main effects of ouabain on immune cells.

Despite the reported preclinical data, several challenges still limit the translational potential of ouabain in GBM therapy. A major obstacle is its poor permeability through the blood–brain barrier (BBB), which may restrict its therapeutic concentrations within the tumor parenchyma (117, 118). Strategies such as nanoencapsulation or chemical modification to improve BBB penetration could help overcome this limitation (119). Another critical aspect is ouabain’s narrow therapeutic index. As a cardiotonic steroid, its systemic use carries the risk of cardiac and metabolic toxicity, emphasizing the need for precise dose control and the development of targeted delivery systems capable of minimizing off-target effects (35, 120). Furthermore, the biological responses elicited by ouabain are highly dose-dependent, ranging from cytotoxic to immunomodulatory (30, 121), underscoring the importance of determining concentration-specific effects in glioma models.

From a therapeutic standpoint, the pleiotropic mechanisms of ouabain suggest that it could act synergistically with current GBM treatments. Its ability to inhibit proliferative and prosurvival signaling pathways may potentiate temozolomide or radiotherapy efficacy, while its immunomodulatory properties could complement immune checkpoint inhibitors by alleviating local immune suppression (30, 122, 123). Future studies should focus on integrating these mechanistic insights into combinatorial approaches, while addressing pharmacological limitations such as BBB permeability and toxicity.

Collectively, these findings explore how ouabain exhibits extensive immunomodulatory action directly or indirectly affects GBM biology. The integration of these mechanisms reinforces ouabain’s relevance as a candidate for therapeutic repurposing. To date, there are no clinical or clinical-stage studies evaluating ouabain or other cardiotonic steroids as therapeutic agents for GBM. All available evidence remains preclinical. This highlights the current insufficiency of translational data and reinforces the need for future studies to address key issues such as efficacy in immunocompetent models, blood–brain barrier permeability, and potential systemic toxicity before any clinical application can be considered.

## 4 Final considerations

**Figure 2:**
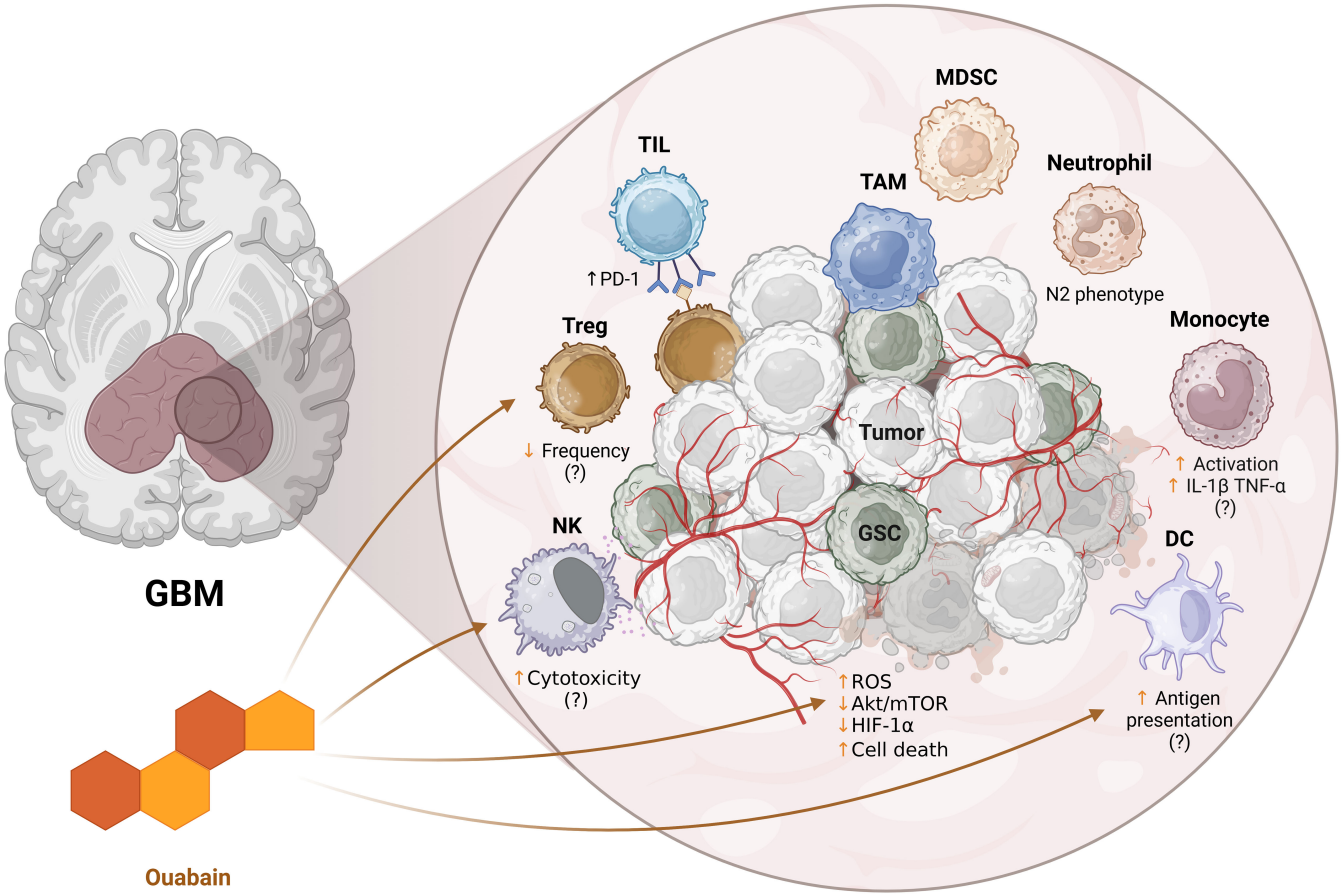
Schematic representation of the potential immunomodulatory effects of ouabain on the tumor microenvironment of glioblastoma (GBM). The enlarged panel on the right details the tumor microenvironment, composed of tumor cells (Tumor), glioma stem cells (GSC), immunosuppressive cells such as TAMs (tumor-associated macrophages), MDSCs (myeloid suppressor cells), Tregs (regulatory T lymphocytes) and neutrophils (e.g.: N2 phenotype), as well as effector cells such as tumor infiltrating lymphocytes (TILs), NK cells and dendritic cells (DCs). Ouabain influences intracellular pathways associated with oxidative stress (ROS), Akt/mTOR pathway, HIF-1α, and tumor cell death. In addition, it appears to modulate different cellular components: NK cell cytotoxicity, Treg frequency, antigen presentation by DCs, and monocyte activation. The suggested effects still lack confirmation (indicated by “?”) and reflect hypotheses based on evidence in other models. ↑ - increase, ↓ - decrease. Created in https://BioRender.com.

Glioblastoma remains one of the most difficult challenges in oncology due to its intrinsic resistance mechanisms and profoundly immunosuppressive microenvironment. Ouabain, a classical cardiotonic steroid, has demonstrated promising antitumor activity in GBM models (**Figure 2**), primarily through the modulation of intracellular signaling pathways, induction of cell death, and inhibition of tumor-promoting processes such as migration and angiogenesis. While direct evidence of its effects on the GBM tumor immune microenvironment remains limited, findings from other biological systems suggest that ouabain may exert broad immunomodulatory effects on both innate and adaptive immunity. These preliminary insights position ouabain as a candidate for therapeutic repurposing in GBM; however, further studies, particularly *in vivo* and within immunocompetent models, are essential to validate its efficacy, define optimal dosing, and understand its impact on immune-tumor dynamics. Future research should also address pharmacological challenges, such as brain barrier permeability and potential systemic toxicity, to enable safe and effective clinical translation.
